# EGFR-TKI resistance promotes immune escape in lung cancer via increased PD-L1 expression

**DOI:** 10.1186/s12943-019-1073-4

**Published:** 2019-11-20

**Authors:** Shunli Peng, Rong Wang, Xiaojuan Zhang, Yueyun Ma, Longhui Zhong, Ke Li, Akihiro Nishiyama, Sachiko Arai, Seiji Yano, Wei Wang

**Affiliations:** 1 Department of Radiation Oncology, Nanfang Hospital, Southern Medical University, Guangzhou, People’s Republic of China; 2 Center for Clinical Medicine Research, Nanfang Hospital, Southern Medical University, Guangzhou, 510515 People’s Republic of China; 3 Divisions of Medical Oncology, Cancer Research Institute, Kanazawa University, Kanazawa, Ishikawa Japan

**Keywords:** PD-L1, EGFR-TKIs resistance, Signaling pathways, Lung cancer, Immunotherapy

## Abstract

**Background:**

The ATLANTIC trial reported that higher PD-L1 expression in tumors was involved in a higher objective response in patients with *EGFR*^*+*^*/ALK*^*+*^ non-small cell lung cancer (NSCLC), indicating the possibility of anti-PD-1/PD-L1 therapy as a third-line (or later) treatment for advanced NSCLC. Therefore, the determination of status and regulatory mechanisms of PD-L1 in *EGFR* mutant NSCLC before and after acquired EGFR-TKIs resistance are meaningful.

**Methods:**

The correlation among PD-L1, c-MET, and HGF was analyzed based on TCGA datasheets and paired NSCLC specimens before and after acquired EGFR-TKI resistance. EGFR-TKI resistant NSCLC cells with three well-known mechanisms, *c-MET* amplification, hepatocyte growth factor (HGF), and *EGFR-T790M*, were investigated to determinate PD-L1 expression status and immune escape ability. PD-L1-deleted EGFR-TKIs sensitive and resistant cells were used to evaluate the immune escape ability of tumors in mice xenograft models.

**Results:**

Positive correlations were found among PD-L1, c-MET, and HGF, based on TCGA datasheets and paired NSCLC specimens. Moreover, the above three resistant mechanisms increased PD-L1 expression and attenuated activation and cytotoxicity of lymphocytes in vitro and in vivo, and downregulation of PD-L1 partially restored the cytotoxicity of lymphocytes. Both MAPK and PI3K pathways were involved in the three types of resistance mechanism-induced PD-L1 overexpression, whereas the NF-kappa B pathway was only involved in *T790M*-induced PD-L1 expression.

**Conclusions:**

HGF, *MET*-amplification, and *EGFR-T790M* upregulate PD-L1 expression in NSCLC and promote the immune escape of tumor cells through different mechanisms.

## Background

Lung cancer is the most common cancer and a leading cause of death from cancer in men and women in the United States [1]. Epidermal growth factor receptor-tyrosine kinase inhibitors (EGFR-TKIs), which are classic small molecule inhibitors used in targeted treatments, have been shown to prolong the survival time of patients with tumours harboring *EGFR*-activating mutations from less than 1 year to approximately 20–30 months [2–5]. Although third-generation EGFR-TKIs could overcome EGFR mutation threonine 790 (T790M) resistance and are becoming the new first-line standard in EGFR mutant non-small cell lung cancer (NSCLC), acquired resistance is virtually inevitable [6]. Multiple resistant mechanisms have been identified, including the activation of c-MET signal pathway [7, 8], MET amplification, hepatocyte growth factor (HGF) and MET overexpression], human epidermal growth factor receptor 2 (HER2) *amplification* [6], and EGFR C797S, L792H and G796R mutations [9]. Among the above mechanisms, high-level MET *amplification* (11–26%), HGF secretion and MET overexpression were frequently detected in EGFR-TKIs resistant NSCLC, especially acquired third generation EGFR-TKIs resistance [10], which indicate that the (MET)/hepatocyte growth factor (HGF) pathway becomes an important resistant mechanism especially in third-generation EGFR-TKIs resistant NSCLC. Therefore, the identification of new therapeutic methods or agents for the treatment of EGFR-TKI resistant lung cancer is imperative.

Immune checkpoint therapy, which is based on negative regulatory mechanisms and targeted enhancement of the anti-tumour immune response [11], is a novel and important therapeutic strategy for lung cancer, especially for patients with advanced non-small-cell lung cancer (NSCLC) [12]. Some retrospective analyses suggest that NSCLC tumours with *EGFR* mutation or anaplastic lymphoma kinase *(ALK*) rearrangements (*EGFR*^*+*^*/ALK*^*+*^) do not respond well to these treatments when compared with *EGFR*^*−*^*/ALK*^*−*^ tumours, indicating that *EGFR* mutant patients are not ideal candidates for anti-PD-1/PD-L1 therapies, compared to patients with *KRAS* mutation or wild-type *EGFR* [13–16]. Recently, the results of the ATLANTIC trial [17, 18] showed the possible efficacy of durvalumab (anti-human PD-1 monoclonal antibodies) as a third-line (or later) treatment for advanced NSCLC, including *EGFR*^*+*^*/ALK*^*+*^ NSCLC. In addition, the PD-L1 expression level in tumour cells may also be involved in the objective responses of patients with *EGFR*^*+*^*/ALK*^*+*^ NSCLC [17, 19]. Moreover, Su et al. [20] reported that one patient with de novo resistance to EGFR-TKIs in addition to PD-L1 and CD8 dual positivity experienced a favorable response to anti-PD-1 therapy. Thus, checkpoint therapies should not be completely excluded from candidate strategies for the treatment of NSCLC patients who acquire resistance to EGFR-TKIs, and unfolding the regulatory mechanisms of PD-L1 in EGFR-TKI resistant NSCLC is thus imperative.

It has been reported that EGFR activation contributed to the upregulation of PD-L1 expression in lung cancers [21], and the expression level of PD-L1 can be decreased by EGFR-TKIs. However, the regulatory mechanisms of PD-L1 and the activity of immune checkpoint inhibitors in EGFR-TKI resistant lung cancer remain unclear. Therefore, we investigated the influence of three important EGFR-TKI resistant mechanisms (HGF, *c-MET* amplification and *EGFR-T790M*) on PD-L1 expression and the immune escape capability of tumours before and after acquired EGFR-TKIs resistance, and explored the regulation mechanisms of PD-L1 in different resistant subtypes.

## Methods

### Cell lines and reagents

The *EGFR* mutant human lung adenocarcinoma cell lines, HCC827 and H1975, were purchased from the American Type Culture Collection (ATCC) Manassas, Virginia, USA. The *EGFR* mutant human lung adenocarcinoma cell line PC-9 was purchased from Immuno Biological Laboratories Co., Ltd., Gunma, Japan. The transfected-human renal derived 293FT cell line was purchased from the Type Culture Collection of the Chinese Academy of Sciences, Shanghai, China. PC-9 and HCC827 cell lines were maintained in RPMI 1640 supplemented medium and the 293FT cell line was maintained in Dulbecco’s modified Eagle’s medium (DMEM). All of the cell lines were cultured as descried previously described [22]. The genome-scale CRISPR-Cas9 knockout lentiviruses (lenti-sgRNA-EGFPMCS, lenti-Cas 9-puro) were used to construct the PD-L1 gene-deleted cell lines and relative negative control cells (PC-9 ^PD-L1-^, PC-9R ^PD-L1-^, PC-9 ^PD-L1+^, and PC-9R ^PD-L1+^) according to the manufacturer’s instructions (Shanghai Genechem Co., Ltd., Shanghai, China).

Gefitinib (a selective EGFR inhibitor), TAK-733 (a selective allosteric inhibitor of MEK), PF-04691502 (an ATP competitive dual PI3K/mTOR inhibitor), and IMD 0354 (an inhibitor of IKKβ) were purchased from Selleck Chemicals (Houston, TX, USA), and dissolved in dimethyl sulfoxide (DMSO, MP Biomedicals, California, USA) at a concentration of 10 mmol/L. Recombinant human HGF Protein was purchased from R&D Systems (Minnesota, USA). Anti-PD-L1 monoclonal antibody (MIH1) was purchased from eBioscience (California, USA).

### Cell growth assay and western blotting

Cell growth assay and western blotting were performed as previously described [23]. Cell growth assay was measured by 3-(4,5-dimethylthiazol-2-yl)-2,5-diphenyl tetrazolium (MTT) assay. Tumor cells (2000/well) were plated into each well of 96-well plates with culture medium. Various concentrations of indicated agents were added to each well 24 h later, and further incubated for 72 h. Then, MTT solution was added and incubated for another 2 h. The media containing MTT solution were removed, and the dark blue crystals were dissolved by adding 100 μL of DMSO. The absorbance was measured with a microplate reader at test and reference wavelengths of 490 and 550 nm. The percentage of growth is shown relative to the untreated controls. Each experiment was done at least in triplicate and thrice independently. Western blotting analysis was performed as described previously [24, 25] and the quantitation results were summarized (Additional file 3). The primary antibodies used in this study included anti-Met (25H2), anti–phospho-Met(Y1234/Y1235), anti-EGFR, anti–phospho-EGFR (Y1068), anti-Akt, anti-phospho-Akt (Ser473), anti-(Erk1/2) (137F5), anti-PD-L1 (#13684) and anti-phospho-p44/42 MAPK (Erk1/2) (Thr202/Tyr204), which were purchased from Cell Signaling Technology, Massachusetts, USA. Anti-human CD274 (PD-L1, B7-H1) antibodies (Function Grade Purified) were purchased from eBioscience, California, USA.

### Flow cytometric analysis

The human CD3 (PE HIT3a), CD4 (PE-cy5 RPA-T4), CD8 (FITC RPA-T4), Hu CD1a (PE), Hu CD83 (FITC), Hu CD16 (FITC), Hu CD56 (PE-Cy7), Hu HLA-DR (APC), and Hu HLA-ABC (FITC) antibodies were purchased from BD Pharmingen. Anti-PD-L1 (E1L3N, 1:400 dilution), anti-rabbit IgG (H + L) secondary antibodies, and F(ab’)2- fragment (Alexa Fluor®555 Conjugate, #4413) were purchased from Cell Signaling Technology. The mean fluorescence intensity (MFI) and mean specific fluorescence intensity (MSFI) were measured to evaluate PD-L1 expression levels, and MSFI was calculated as the ratio of the MFI of anti-PD-L1 antibody to that of the control antibody [26]. Each experiment was performed at least in triplicate, and thrice independently.

### Isolation and activation of human PBMC and T lymphocytes

Peripheral blood mononuclear cells (PBMCs) were isolated using density-gradient-centrifugation-(Ficoll), and the activation of T cells was performed as previously described [27, 28]. Briefly, PBMCs were isolated according to the manufacturer’s instructions and then cultured in RPMI 1640 and 10% FBS overnight. Then, the supernatant T cells were collected and stimulated for 2 days with PHA (10 μg/mL) and rhIL-2 (4000 UI/mL) to promote the proliferation and activation of T lymphocytes. Finally, T lymphocytes were cultured with rhIL-2 (2000 UI/mL) in RPMI 1640 and 10% FBS to obtain the survival of activated T lymphocytes.

### Xenograft studies in NOD-SCID mice

The in vivo experimental project was approved by Nanfang Hospital Animal Ethic Committee (NFYY-2016-63), and four-week-old male NOD-SCID mice that were used in the in vivo experiment were purchased and maintained in a specific pathogen-free (SPF) institution of experimental animal center of Nanfang Hospital, Guangzhou, China. Firstly, four-week-old male NOD-SCID mice were randomly assigned into 4 groups, and PC-9^PD-L1-^, PC-9R^PD-L1-^, PC-9^PD-L1+^, and PC-9R^PD-L1+^ cells (4 × 10^6^/ per xenograft) were injected subcutaneously to establish 4 xenograft tumour models (*N* = 8). Then, each xenograft tumour model was randomly assigned into 2 group (*N* = 4) with intraperitoneal injection of phosphate buffer saline (PBS) or human immunocyte mixtures consisting of human PBMC (5 × 10^6^/per mouse) and activated T lymphocytes (1 × 10^7^*/per mouse*) after tumour injection on days 0, 7, 14, and 21.Tumour volumes were measured with Vernier calipers along their width (a) and length (b), and tumour volume was calculated using the eq. TV = (a^2^b)/2. Tumor regression rate = (Tumour volume in NOD-SCID mice after immunocyte mixtures treatment / tumour volume in NOD-SCID mice after PBS treatment) × 100%. All of the mice were housed in a pathogen-free environment.

### Specimens and TCGA data

In total, 16 tumour specimens with EGFR-activating mutations were provided by the Kanazawa University Hospital (Kanazawa, Japan). Detailed patient information for the EGFR-TKI resistant specimens is described in Additional file 2: Table S1. The TCGA datasets of lung adenocarcinoma (PanCancer Atlas and provisional) were retrieved from cBioProtal (http://www.cbioportal.org/study?id=luad_tcga#summary). The detailed analysis is provided in the Additional file 1: Supplementary materials and methods.

### Statistical analysis

All data were presented as the mean ± standard deviation (SD), and differences between the means were examined by student’s t test or one-way ANOVA using statistical software (SPSS, version 20, IBM Corp., Armonk, USA). Differences with a value of *P* < 0.05 were considered statistically significant. All of the experiments were performed at least thrice.

## Results

### Changes in PD-L1 expression after acquiring EGFR-TKI resistance in NSCLC

**Fig. 1:**
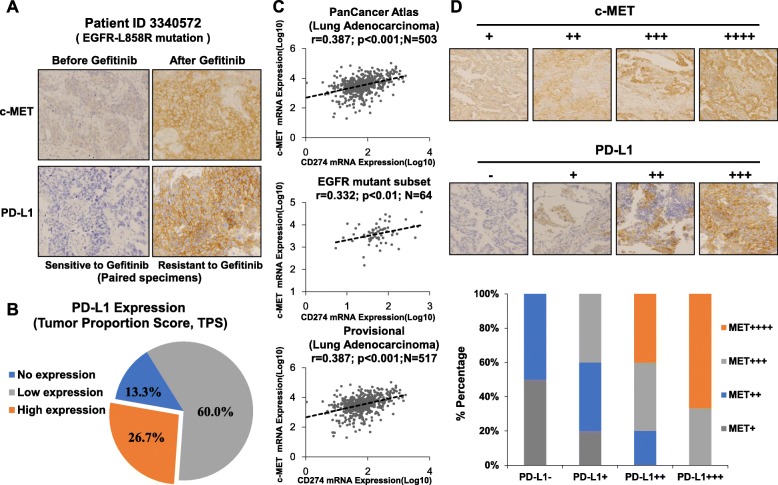
The expression of PD-L1 in NSCLC changes after acquisition of EGFR-TKIs resistance based on the patient biopsy specimens and TCGA datasets. **a** PD-L1 expression increased in a subgroup of EGFR-TKI resistant specimens. PD-L1 and c-MET expression status in paired specimens before and after acquired gefitnib resistance were measured by IHC staining. **b** PD-L1 expression status in EGFR-TKI resistant NSCLC specimens (*N* = 15). **c** and **d** The correlation coefficient between PD-L1 (CD274) and c-MET in lung adenocarcinoma and *EGFR* mutant subsets based on the TCGA datasets (Lung Adenocarcinoma, PanCancer Atlas and Provisional) and in EGFR-TKI resistant NSCLC specimens

Based on the paired specimens from the same patient (*EGFR-L858R* mutation) who initially benefitted from gefitinib treatment and then acquired resistance (Fig. 1a), we investigated the change in tumours before and after acquired EGFR-TKI resistance. The results showed that PD-L1 expression in specimens increased from < 1% to ≥50% after the patient acquired gefitinib resistance. Moreover, in the resistant specimens, *EGFR-T790M* mutation could not be detected, whereas high c-MET expression was measured (Fig. 1a, Additional file 2: Table S1). To clarify the expression status of PD-L1 in EGFR-TKIs resistant NSCLC and determine why PD-L1 expression in the tumours of these patients changed after acquired EGFR-TKIs resistance, we collected specimens from 15 NSCLC patients who acquired EGFR-TKI resistance (Additional file 2: Table S1) and detected PD-L1 expression via PD-L1 IHC 22C3 pharmDx. Figure 1b shows that the percentage of no, low, or high PD-L1 expression among the 15 EGFR-TKI resistant specimens was 13.3, 60 and 26.7%, respectively. These results suggest that a subgroup of EGFR-TKI resistant NSCLC tumours expresses a high level of PD-L1.

Since we found that *MET*^*+*^ mutant NSCLC patients seem to have higher PD-1, PD-L1 expression compared with the *EGFR*^*+*^ and *KRAS*^*+*^ mutant subgroups (Additional file 1: Figure S1), and high expression of c-MET and PD-L1 were detected in gefitinib resistant specimens (Fig. 1a), we investigated whether the upregulation of PD-L1 in these patients was related to the c-MET activation. Thus, we analyzed the correlation between *PD-L1* (CD274) and *c-MET* based on two TCGA datasets (Lung Adenocarcinoma, PanCancer Atlas, provisional). As shown in Fig. 1c, the coefficient of correlation (R^2^) of *PD-L1* and *c-MET* expression in lung adenocarcinoma or the *EGFR* mutant subgroup was 0.332–0.387 (*p* < 0.05, Fig. 1c). To further investigate the correlation between PD-L1 and c-MET in EGFR-TKI resistant NSCLC, we measured the PD-L1 and c-MET expression in 15 EGFR-TKI resistant biopsies. The results revealed a higher percentage of c-MET expression in specimens with high expression of PD-L1; the coefficient of correlation between PD-L1 and c-MET was 0.591 (*p* < 0.05, Fig. 1d), indicating a positive correlation between PD-L1 and c-MET in EGFR-TKI resistant NSCLC.

### HGF induces PD-L1 expression in *EGFR*-mutant NSCLC cells and moderates proliferation and cytotoxicity of T lymphocytes

**Fig. 2:**
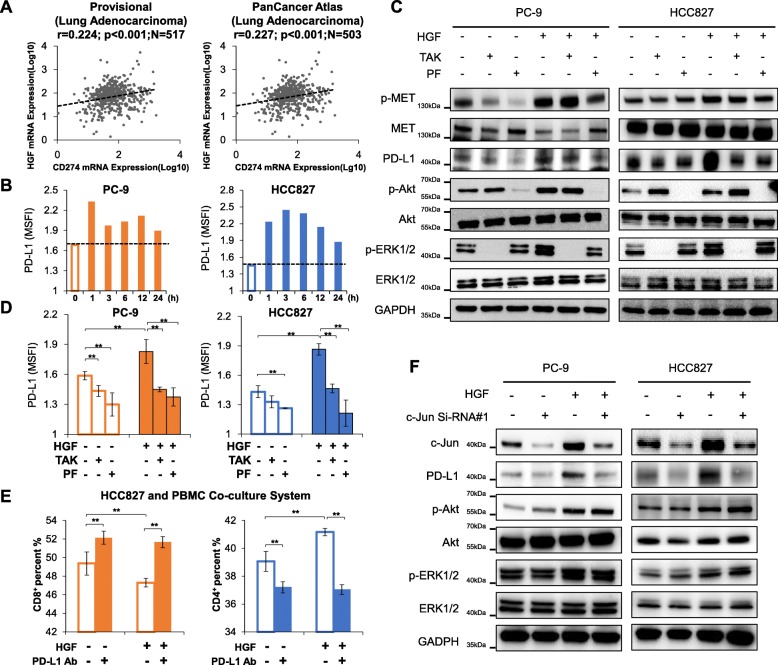
HGF upregulates PD-L1 expression by activating PI3K/Akt and MAPK signaling pathway, but not NF-kappa B signaling pathway to promote the phosphorylation and activation of AP-1 in *EGFR* mutant NSCLC cells. **a** The correlation coefficient between PD-L1 (CD274) and HGF in lung adenocarcinoma based on the TCGA datasets (Lung Adenocarcinoma, PanCancer Atlas, and Provisional). * *P* < 0.05; ** P < 0.01. **b** PC-9 and HCC827 cells were treated with HGF (50 ng/L) for different periods of time, then harvested and measured by flow cytometry to evaluate the expression of PD-L1. **c** and **d** PC-9 and HCC827 cells were pre-treated with TAK-733 (TAK, 0.3 μmol/L) or PF-04691502 (PF, 0.3 μmol/L) for 6 h, then stimulated with HGF (50 ng/L) or PBS for an additional 6 h. Cells were harvested and analyzed by western blotting and flow cytometry. Bars indicate SD,* *P* < 0.05; ** P < 0.01. **e** HGF moderates the activation and cytotoxic effect of T lymphocytes, and anti-PD-L1 antibodies restores their cytotoxicity. HCC827 cells were pretreated with or without HGF (50 ng/L), anti-PD-L1 antibodies (10 μg/mL) or control IgG for an hour. Then all the cells were co-cultured with human PBMC for 72 h at an effector/target cell ratio = 6:1. The percentage of CD3^+^/CD4^+^, CD3^+^/CD8^+^ cells in the co-culture system was analysed by flow cytometry. **f** PC-9 and HCC827 cells were transfected with *c-Jun* Si-RNA for 72 h, then stimulated with HGF (50 ng/L) or PBS for an additional 6 h. Cells were harvested and analysed by Western blotting. All experiments were performed at least three times

HGF not only activates the c-MET signaling pathway to induce EGFR-TKIs resistance in lung adenocarcinomas [23, 29], but it also promotes the transcription of the endogenous *c-MET* gene [30]. Based on the positive correlation between PD-L1 and c-MET expression (Fig. 1c-d), we investigated whether HGF influences PD-L1 expression in EGFR-TKI resistant NSCLC. Based on the two TCGA datasets (Lung Adenocarcinoma, PanCancer Atlas and Provisional), the coefficient of correlation between HGF and PD-L1 expression was 0.224, and 0.227, respectively (*p* < 0.01) (Fig. 2a). To further confirm the influence of HGF on PD-L1 in EGFR-TKI resistant NSCLC, we added HGF (50 ng/L) to the culture medium of *EGFR* mutant NSCLC cells (PC-9, HCC827) to establish the HGF-mediated EGFR-TKI resistant NSCLC cell models. The results showed that PD-L1 expression was increased after stimulation of HGF (50 ng/L, Fig. 2b-d, Additional file 1: Figure S2). Moreover, because the PD-1/PD-L1 axis promotes tumour cell escape from immune surveillance by inhibiting the proliferation, activation and cytotoxicity of T lymphocytes [31], we investigated whether HGF-induced upregulation of PD-L1 moderates the proliferation and cytotoxicity of T cells in vitro. Peripheral blood mononuclear cells (PBMC) cells of healthy donators were collected and cultured with tumour cells (HCC827) to evaluate the percentage and activation of CD8^+^ T cells. As shown in Additional file 1: Figure S3A, the percentage of dendritic cells (DCs) in PBMCs was 4–5%, indicating the capacity of antigen presentation to naïve T cells. As expected, the percentage of CD8^+^ T cells in PBMCs increased after co-culturing with tumour cells, indicating the proliferation and activation of anti-tumour specific T cells (Additional file 1: Figure S3B and C). HGF did not change HLA-ABC expression in HCC827 cells (Additional file 1: Figure S3D). However, treatment with HGF decreased the percentage of CD8^+^ T cells and the secretion of IFN-γ in the co-culture system (Fig. 2e, Additional file 1: Figure S4A), indicating that HGF weakens the cytotoxicity of T cells toward tumours. In addition, anti-PD-L1 antibodies partially increased the percentage of CD8^+^ T cells and the secretion of IFN-γ in the co-culture system. The percentage of CD3^−^CD16^+^CD56^+^ NK cells in PBMCs is approximately 1% (Additional file 1: Figure S4B) with undetectable cytotoxicity to PC-9 or HCC827 cells in the co-culture system (data not shown). These results suggest that HGF may promote immune escape of tumour cells via overexpression of PD-L1 in EGFR-TKI resistant NSCLC cells.

### HGF induces PD-L1 expression in NSCLC cells by activation of PI3K/Akt, MAPK, and AP-1 (activator protein 1)

Because HGF may increase EGFR-TKI resistance, PD-L1 expression, and immune escape in NSCLC, we explored the regulatory mechanisms of PD-L1 expression in HGF-mediated EGFR-TKI resistant NSCLC tumours. As the phosphoinositide 3-kinase (PI3K)/Akt and mitogen-activated protein kinase (MAPK) signaling pathways are critical to EGFR-TKIs resistance of *EGFR* mutant NSCLC cells [23] and the NF-kappa B pathway is reported to be activated by HGF in many tissues [32, 33], we investigated whether these three pathways are involved in HGF-mediated PD-L1 expression. Consistent with our previous reports [29, 34], both the PI3K/Akt and MAPK signaling pathways were activated after treatment with an HGF, PI3K inhibitor (PF-04691502) and MEK inhibitor (TAK733), and successfully inhibited the phosphorylation of Akt and ERK1/2 (Fig. 2c). Moreover, PD-L1 expression increased after stimulation of HGF, while inhibiting PI3K/Akt or MAPK signal pathway abolished HGF induced-PD-L1 upregulation (Fig. 2c-d). However, inhibition of NF-kappa B pathway had no significant influence on PD-L1 expression after stimulation of HGF (Additional file 1: Figure S5).

Activator protein (AP-1), a transcription factor that consists of a variety of dimers composed of members including c-Jun and c-FOS, has been reported to be activated by the PI3K and MAPK signaling pathways and could promote transcription of PD-L1 in melanoma cells [35]. Therefore, we hypothesized that AP-1 is involved in HGF-induced PD-L1 expression in lung cancer. Figure 2f shows that the knockdown of c-Jun decreased HGF-induced PD-L1 expression, even though the PI3K/Akt and MAPK pathways were activated. These results suggest that the PI3K/Akt, MAPK pathways, and AP-1 are involved in HGF-induced PD-L1 upregulation in EGFR mutant NSCLC.

### *c-MET* amplification mediated-EGFR-TKIs resistance upregulates PD-L1 expression and promotes immune escape ability of *EGFR*-mutant NSCLC cells

**Fig. 3:**
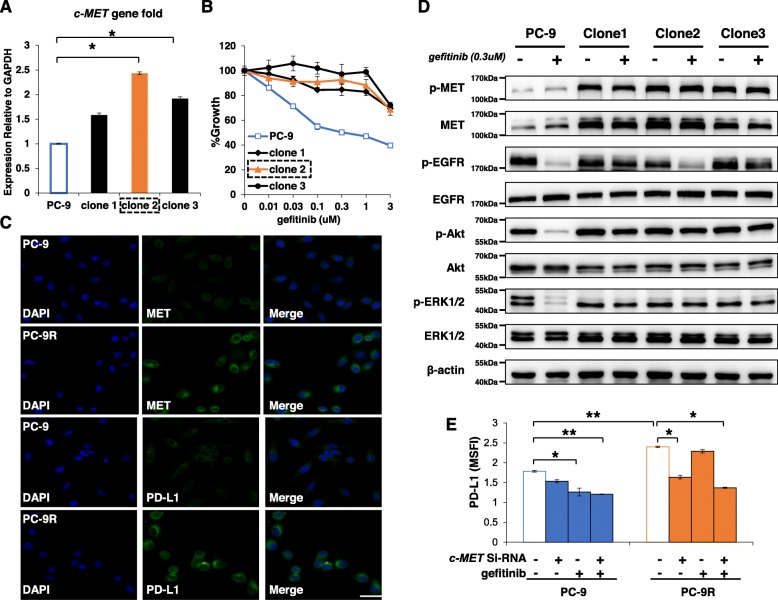
*c-MET* amplification upregulated PD-L1 expression in *EGFR*-TKI resistant NSCLC cells. **a** Three clones were picked from gefitinib-resistant PC-9 cells, and incubated with increasing concentrations of gefitinib (1 μmol/L, final concentration) over 6 months. *c-MET* amplification was measured by RT-qPCR. Clone 2 was named PC-9R and used for further study. Bars indicate SD.* *P* < 0.05. **b** PC-9 and the three clones were treated with various concentrations of gefitinib, and cell growth was determined after 72 h of stimulation using the MTT assay. **c** Expression of c-MET and PD-L1 protein in PC-9 and PC-9R cells was measured by immunofluorescence staining and visualized using a laser confocal microscopy (× 400) (bars = 200 μm). **d** PC-9 cells and three clone cells were treated with gefitinib (1 μmol/L) for 12 h, and the lysates were harvested. Indicated proteins were determined by western blotting. **e** PC-9 and PC-9R cells were treated with or without *c-MET* siRNA for 48 h, and cultured with or without treatment of gefitinib (1 μmol/L). After further 12 h, cells were collected and PD-L1 expression was examined by flow cytometry. All of the experiments were performed at least thrice

As *c-MET* amplification increases c-MET expression [36], we explored whether *c-MET* amplification could increase the expression of PD-L1. We generated *c-MET*-amplified EGFR-TKI resistant clones by exposing *EGFR* mutant NSCLC cells (PC-9) to increasing concentrations of gefitinib over 6 months as previously described [36, 37]. Three clones from the gefitinib-resistant PC-9 cells showed *c-MET* amplification and acquired resistance to gefitinib (Fig. 3a-c). Clone 2 (named PC-9R) was selected for subsequent experiments due to the high expression of the *c-MET* gene. Figure 3d and Additional file 1: Figure S6 show that c-MET and PD-L1 expression in PC-9R cells was higher than in PC-9 cells. Moreover, gefitinib successfully decreased PD-L1 expression in PC-9 cells but had no significant influence on PD-L1 expression on PC-9R cell. In contrast, *c-MET* siRNA effectively decreased PD-L1 expression in PC-9R cells (Fig. 3e, Additional file 1: Figure S7). In both PC-9 and PC-9R cells, *c-MET* siRNA combined with gefitinib effectively downregulated PD-L1 expression. These results suggest that *c-MET* amplification upregulates PD-L1 expression in EGFR-TKI resistant NSCLC cells.

**Fig. 4:**
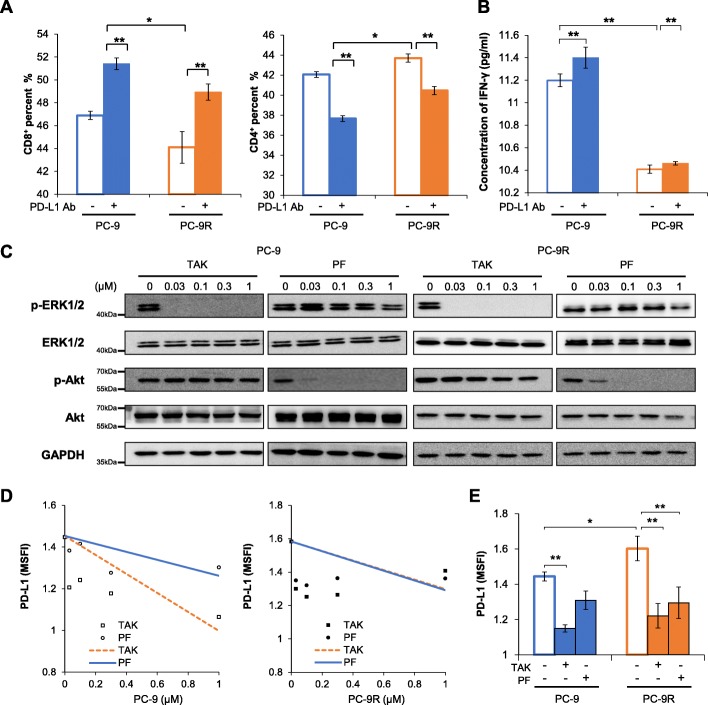
**a** *c-MET* amplification mediates PD-L1 moderated the activation and cytotoxic effect of T lymphocytes, and anti-PD-L1 antibodies restored the cytotoxicity of T lymphocytes. PC-9 or PC-9R cells were pretreated with anti-PD-L1 antibodies (10 μg/mL) or control IgG for an hour, then co-cultured with human PBMC for a further 72 h at an effector/target cell ratio = 6:1. The percentage of CD3^+^/CD4^+^, CD3^+^/CD8^+^ cells in the co-culture system was analyzed by flow cytometry. **b** IFN-γ concentration in the supernatant of co-culture systems. PC-9 or PC-9R cells were pre-treated with anti-PD-L1 antibodies (10 μg/mL) or control IgG for an hour, then co-cultured with human PBMC for further 72 h at an effector/target cell ratio = 6:1. The concentration of IFN-γ in the supernatant was detected by human IFN-γ ELISA assay (multi sciences company, EK1802). Bars indicate SD; ** *P* < 0.01.**c**-**e** PI3K/Akt and MAPK signaling pathways are involved in *c-MET* amplification mediated upregulation of PD-L1 in EGFR-TKI resistant lung cancer. **c** and **d** TAK-733 (MEK inhibitor) and PF-04691502 (dual PI3K/mTOR inhibitor) successfully inhibited the MAPK and PI3K signaling pathways. PC-9 and PC-9R cells were incubated with various concentrations of TAK-733 or PF-04691502 in concentration of 0, 0.01, 0.1, 0.3, 1 μmol/L respectively. After 12 h, the cells were harvested and analysed by western blotting and flow cytometry. **e** PC-9 and PC-9R cells were incubated with TAK-733 (0.3 μmol/L) or PF-04691502 (0.3 μmol/L) for 12 h, then harvested and analyzed by flow cytometry. Bars indicate SD. * *P* < 0.05, ** *P* < 0.01. All of the experiments were performed at least thrice

To evaluate the immune escape capability of PC-9 and PC-9R cells, we co-cultured PBMC cells with PC-9 or PC-9R cells and found that the percentage of CD8^+^ T cells and secretion level of IFN-γ was lower in the presence of PC-9R, although PC-9 and PC-9R cells expressed similar levels of HLA-ABC (Fig. 4a-b, Additional file 1: Figure S8). In addition, anti-PD-L1 antibodies increased percentage of CD3^+^/CD8^+^ cells (CD8^+^ T cells) and secretion of IFN-γ in both co-culture systems. These results suggest that *c-MET* amplification may promote the immune escape of EGFR-TKI resistant NSCLC cells partially through overexpression PD-L1.

### PI3K/Akt and MAPK, but not NF-kappa B, signaling pathways are involved in *c-MET* amplification-induced upregulation of PD-L1 in EGFR-TKIs resistant NSCLC cells

We investigated whether PI3K, MAPK, and NF-kappa B signaling pathways participate in the upregulation of PD-L1 mediated by *c-MET* amplification. As shown in Fig. 4c-e, TAK733 seems to be more effective than PF-04691502 in PD-L1 downregulation in PC-9 cells, which means that the PI3K/Akt pathway may be less important than the MAPK signaling pathway in the regulation of PD-L1 in gefitinib-sensitive cells. However, both PF-04691502 and TAK733 effectively decrease PD-L1 expression in PC-9R cells, which indicates that both PI3K/Akt and MAPK pathways seem to be critical after acquired gefitinib resistance mediated by *c-MET* amplification. Moreover, inhibition of the NF-kappa B pathway downregulated PD-L1 expression in PC-9 cells but not PC-9R cells (Additional file 1: Figure S9), suggesting that the NF-kappa B pathway is less important in *c-MET* amplification-induced PD-L1 expression, even though the NF-kappa B pathway is associated with *EGFR*-induced PD-L1 expression [38]. Taken together, these results suggest that the PI3K/Akt and MAPK, but not the NF-kappa B, signaling pathways are critical in the upregulation of PD-L1 induced by *c-MET* amplification in EGFR-TKI resistant NSCLC cells.

### *EGFR-T790M* mutation upregulates PD-L1 expression through the PI3K/Akt, MAPK, and NF-kappa B signaling pathways

**Fig. 5:**
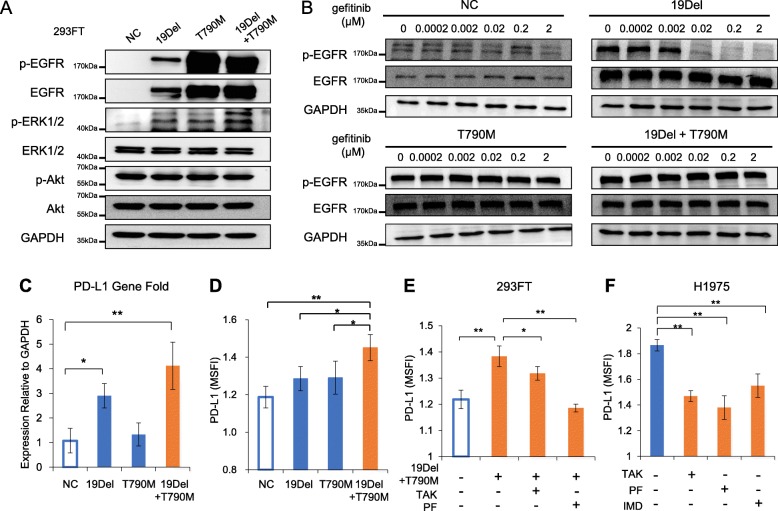
*EGFR-T790M* secondary mutation upregulates PD-L1 expression in tumour cells through the PI3K/Akt, MAPK, and NF-kappa B signal pathways. **a**-**d** 293FT cells were transfected with control vector plasmid (NC), *EGFR-19Del* (*19Del*), or *EGFR-T790M* (*T790M*) mutation plasmids for 48–72 h, then treated with/without gefitinib for a further 24 h. All the cells were harvested and analyzed by western blotting, RT-qPCR and flow cytometry. Bars indicate SD. * *P* < 0.05; ** P < 0.01. **e** 293FT cells were transfected as described above for 48 h, and then treated with TAK-733 (TAK, 0.3 μmol/L) or PF-04691502 (PF, 0.3 μmol/L) for an additional 12 h. PD-L1 expression was evaluated by flow cytometry. Bars indicate SD. * *P* < 0.05; ** P < 0.01. **f** H1975 cells were treated with TAK-733(0.3 μmol/L), PF-04691502(0.3 μmol/L), or IMD 0354 (IMD, 1 μmol/L) for 24 h and PD-L1 expression was evaluated by flow cytometry. Bars indicate SD. * *P* < 0.05; ** P < 0.01. All experiments were performed at least thrice

*EGFR-T790M* mutation (*T790M*) accounts for the greatest number of cases of acquired resistance to first generation EGFR-TKIs. To investigate the relationship between *T790M* and PD-L1, we transfected the *EGFR-exon 19 deletion* (*19Del*) or *EGFR-T790M* mutation into 293FT cells to mimic the status of NSCLC before and after developing resistance to EGFR-TKIs via *T790M* mutation. Figure 5a and b show that the transfection of *19Del* and/or *T790M* induced a high level of EGFR activation, and gefitinib effectively inhibited the phosphorylation of EGFR in the cells transfected by *19Del*, but not *T790M* or *19Del/T790M*. Interestingly, PD-L1 expression increased in 293FT cells that were transfected with *19Del*, whereas transfection of *19Del/T790M* resulted in a higher level of PD-L1 expression (Fig. 5c and d, and Additional file 1: Figure S10). Moreover, PI3K and MAPK pathway inhibition reduced PD-L1 expression in both 293FT cells transfected with *19Del/T790M* mutation and H1975 cells containing *EGFR-T790M* mutation (Fig. 5e and f). Unlike the mechanisms of *c-MET* amplification and HGF on the regulation of PD-L1, *T790M*-induced PD-L1 expression was suppressed after inhibition of the NF-kappa B pathway (Fig. 5e). These results suggest that the PI3K, MAPK, and NF-kappa B pathways are all involved in the regulation of PD-L1 in *EGFR-T790M* mutation-mediated EGFR-TKI resistant NSCLC cells.

### Restoration of the cytotoxic effect of human T lymphocytes in vivo via the downregulation of PD-L1 expression in EGFR-TKI resistant lung cancer

**Fig. 6:**
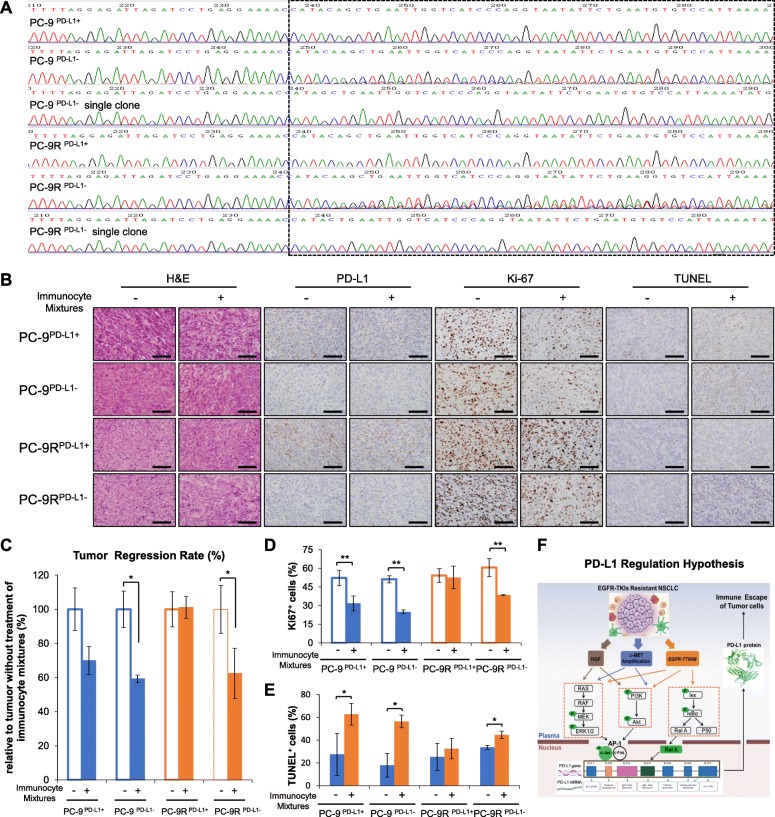
Downregulation of PD-L1 expression in EGFR-TKI resistant lung cancer restores the cytotoxic effect of human T lymphocytes in vivo*.* **a** Establishment of PD-L1 gene knockout cell lines. CRISPR-Cas9 knockout lentiviruses were used to delete the PD-L1 gene on a genome-wide scale. Control cells were named PC-9^PD-L1+^ and PC-9R ^PD-L1+^, and PD-L1 gene deletion knockout cells were designated as PC-9^PD-L1-^ or PC-9R^PD-L1-^. The sequencing results of the above cells are shown to evaluate the knockdown effect. **b** H&E staining, IHC staining and TUNEL assay results of PC-9 ^PD-L1+^, PC-9R ^PD-L1+^, PC-9 ^PD-L1-^, PC-9R ^PD-L1-^ xenografts (× 400) with/without treatment of immunocyte mixtures or PBS (bars = 200 μm). **c** The cytotoxic effect of the immunocyte mixtures was evaluated by calculating xenografts volumes in PC-9 ^PD-L1+^, PC-9R ^PD-L1+^, PC-9 ^PD-L1-^, PC-9R ^PD-L1-^ tumour xenografts, and the treatment effects are shown in the bar chart. Bars indicate standard error (SD). * *P <* 0.05. **d**-**e** Histograms showing the percentage of Ki-67^+^ or TUNEL^+^ cells in xenografts. Bars indicate SD. * *P* < 0.05; ** P < 0.01*.* **f** Different regulator models of PD-L1 in different subgroups of EGFR-TKI resistant NSCLC

To compare the immune escape capability of tumour cells among EGFR-TKI sensitive and resistant NSCLC in vivo, we generated EGFR-TKI sensitive and resistant NSCLC cells with *PD-L1* gene deletion, and its control cells (PC-9^PD-L1-^, PC-9R^PD-L1-^, PC-9^PD-L1+^, and PC-9R^PD-L1+^) using the CRISPR-Cas9 knockout lentivirus. We confirmed the base sequence of the *PD-L1* gene in the cells to confirm knockdown efficacy (Fig. 6a). The PC-9 and PC-9R cells exhibited the same sensitivity to EGFR-TKIs after deletion or overexpression of the *PD-L1* gene (Additional file 1: FiguresS11-S12). Meanwhile, overexpression PD-L1 has no influence on EGFR expression (Additional file 1: Figure S12B). Then, all four cells were injected subcutaneously in NOD-SCID mice to build the xenograft models described in materials and methods. In addition, human immunocyte mixtures rather than PBMC [39] were injected into the NOD-SCID mice to kill the xenografts more effectively. Figure 6b shows that low PD-L1 expression in PC-9^PD-L1-^ and PC-9R^PD-L1-^ tumours shows the effectiveness of PD-L1 gene deletion. Moreover, tumours in PC-9^PD-L1+^ group were degraded after treatment with human immunocyte mixtures, however tumours in the PC-9R^PD-L1+^ group did not respond to the treatment, suggesting an immune suppression environment in PC-9R^PD-L1+^ tumours. With respect to the PD-L1 gene deletion groups, the tumours in the PC-9^PD-L1-^ and PC-9R^PD-L1-^ groups shrank remarkably, suggesting an anti-tumour effect of T cells restored after PD-L1 depletion on tumour cells (Fig. 6c). Owing to the short survival time in NOD-SCID mice, the percentage and distribution of human CD8+ T cells and other human immune cells could not be detected in vivo after treatment with human immunocyte mixtures for more than 1 month.

The results of H&E staining indicated no significant morphologic abnormalities in any group. In addition, the percentage of Ki67^+^ cells and apoptotic cells (Fig. 6d-e) indicated that immunocyte mixtures treatment decreased the proliferation of tumour cells (Ki67-positive) and promoted apoptosis of tumour cells (TUNEL positive) in the PC-9 groups (PC-9^PD-L1+^ and PC-9^PD-L1-^). Alternatively, there was no significant influence on the PC-9R^PD-L1+^ tumours, indicating immune depression tumour microenvironment (TME) on xenografts in vivo after acquired EGFR-TKIs resistance. In contrast, PC-9R^PD-L1-^ tumours responded to the treatment with significantly lower percentage of Ki67 positive cells and a higher percentage of TUNEL-positive cells. These results clearly showed that overexpression of PD-L1 in PC-9R cells mediated the immune escape of tumour cells, whereas PD-L1 depletion successfully restored the cytotoxicity of lymphocytes to PC-9R cells in vivo.

## Discussion

The PD-1/PD-L1 axis plays a prominent role in immune suppression of the tumour microenvironment [40, 41], and patients with higher tumour PD-L1 expression achieve improved responses to treatment with anti-PD-1 and anti-PD-L1 therapy, compared with patients with lower PD-L1 expression. Thus, determining the regulatory mechanisms of PD-L1 in tumours is meaningful and necessary. Recently, PD-L1 was found to increase after the development of resistance to BRAF inhibitors [35]. However, very few studies have examined the connection between EGFR-TKI resistance and PD-L1 expression in lung cancer. In this study, the paired EGFR-TKI sensitive and resistant specimens and TCGA datasets suggested that PD-L1 expression may be different after acquisition of EGFR-TKI resistance, and PD-L1 expression was found to be positively correlated to c-MET expression. Our data suggest that HGF, *c-MET amplification*, and *EGFR T790M* mutation can upregulate PD-L1 expression and promote immune escape capability in NSCLC, and that the relative regulatory mechanisms of PD-L1 expression among three subtypes may be vary. Other EGFR-TKI resistant mechanisms, such as EMT, *PTEN* loss, and activation of IGF-1R, should be further investigated to determine their influence on PD-L1 expression on EGFR-TKIs resistant NSCLC.

The PI3K/Akt and MAPK signaling pathways are widely reported to participate in the regulation of PD-L1 in many tumours, and NF-kappa B was found to be involved in the EGFR-TKI induced downregulation of PD-L1 in *EGFR*-mutant NSCLC [38]. In our study, we found that the PI3K-Akt and MAPK signaling pathways are both involved in the overexpression of PD-L1 induced by *c-MET amplification*, the HGF/c-MET axis, and *EGFR-T790M* mutation. We further identified that AP-1 may be involved in HGF-induced PD-L1 expression. Alternatively, the NF-kappa B pathway was involved in *EGFR-T790M* mutation-induced PD-L1 expression and had no significant influence on PD-L1 expression induced by HGF and *c-MET amplification* (Fig. 5f; Additional file 1: Figure S5, S9). This suggests that the NF-kappa B pathway is largely involved in *EGFR*-related PD-L1 expression, and less important in bypass resistance induced-PD-L1 expression. These results suggest that PD-L1 regulation in NSCLC with different EGFR-TKI resistant mechanisms may not be the same. Moreover, we infer that some EGFR-TKI resistant mechanisms related to the abnormal activation of the PI3K/Akt pathway, such as *PTEN* loss, may also increase PD-L1 expression.

EGFR-TKI treatment is the first-line therapy for *EGFR* mutant NSCLC. However, whether immunotherapy may be used in the treatment of EGFR-TKI resistant *EGFR* mutant NSCLC is yet to be determined. Previous studies have shown that *EGFR* mutations are associated with a low response rate to PD-1/PD-L1 inhibitors in NSCLC, and objective responses were observed in 1 of 28 (3.6%) EGFR-mutant patients versus 7 of 30 (23.3%) EGFR wild-type patients [15]. However, in patients with *EGFR* mutation, the objective response rate (ORR) of immunotherapy in the PD-L1 high expression group was 12.2%, while it was only 3.6% in the PD-L1 low expression group [42]. Haratani [43] found that *T790M*-negative patients with *EGFR* mutation-positive NSCLC are more likely to benefit from Nivolumab (anti-human PD-1 monoclonal antibodies) after EGFR-TKI treatment, possibly as a result of a higher PD-L1 expression level, when compared with *T790M*-positive patients, which indicates that patients with other resistance mechanisms, such as MET activation, may have higher response rates compared with those with *T790M* mutations in NSCLC. Therefore, despite the lower response of PD-1/PD-L1 therapy in *EGFR* mutant compared with EGFR wild-type patients, higher PD-L1 expression still indicates a better response to anti-PD-1/PD-L1 inhibitors in *EGFR* mutant advanced lung cancer patients who acquired EGFR-TKI resistance. In addition, PD-L1 expression and the response rate may be different among EGFR-TKI resistant advanced NSCLC patients. In our study, we found that some EGFR-TKI mechanisms may upregulate PD-L1 expression and promote the immune escape ability of EGFR-TKIs resistant NSCLC tumours, but it does not indicate an absolute response to anti-PD-1/PD-L1 therapies. A series of factors, such as tumour mutation burden (TMB), co-occurring genomic alterations, microsatellite instability (MSI), tumour-infiltrating lymphocytes (TILs), and immunogenic/no-immunogenic (hot or cold) TME [11, 12, 44–48], have been considered as predictors of the response rate of patients to immune checkpoint therapies, i.e., that the efficacy of immune checkpoint therapies could be influenced by several factors. In our study, we found that TMB, PD-1 and PD-L1 expression in *EGFR*, *KRAS*, and *c-MET* mutant NSCLC might be vary (Additional file 1: Figure S1). Based on the PanCancer Atlas datasets, the mRNA expression level of PD-1 and PD-L1 in *c-MET* mutant NSCLC was higher than that with *EGFR* mutation. Therefore, whether the TME of *EGFR*^*+*^*/c-MET*^*+*^ mutant NSCLC, such as EGFR-TKI resistant NSCLC acquired *c-MET amplification*, is different from the *EGFR*^*+*^*/c-MET*^*−*^ mutant NSCLC, and the response rates to PD-1/PD-L1 therapies require further investigation. Recently, the IMpower150 trial observed an improved overall survival with atezolizumab plus bevacizumab plus carboplatin plus paclitaxel (ABCP) versus bevacizumab plus carboplatin plus paclitaxel (BCP) in patients with sensitizing EGFR mutations, which shows a potential role of antiangiogenic drugs in enhancing the efficacy of immunotherapy in EGFR mutated patients [49]. Thus, whether EGFR-TKI resistant patients could ultimately benefit from checkpoint therapies and the best combination models among checkpoint therapy, chemotherapy, radiotherapy and targeted therapy need to be further investigated.

## Conclusions

We found that acquired EGFR-TKI resistance promotes the immune escape in lung cancer by upregulating PD-L1 expression. The PI3K-Akt, MAPK, and NF-kappa B signaling pathways and AP-1 are involved in the upregulation of PD-L1 induced by different EGFR-TKI resistant mechanisms. The results of our research may partially explain the different PD-L1 status in EGFR-TKI sensitive and resistant tumours and unveil the regulatory mechanisms of PD-L1 in EGFR-TKI resistant NSCLC. Our study provides insights into PD-L1 expression in the different subgroups of EGFR-TKI resistant NSCLC and may have specific implications for the possibility of immune-checkpoint therapy in different subgroups of EGFR-TKI resistant NSCLC.

## Supplementary information

**Additional file 1:** **Figure S1.** The tumor mutation burden (TMB), PD-L1, PD-1 and CTLA-4 expression in *EGFR*, *c-MET* and *KRAS* mutant subgroups basing on TCGA Datasheet (Lung Adenocarcinoma, PanCancer Atlas). **Figure S2.** PD-L1 Expression after stimulation of HGF. **Figure S3.** A. The percentage of DC cells in PBMC separated from health donators. **Figure S4.** A. IFN-γ concentration in the supernatant of co-culture systems. **Figure S5.** Inhibition of NF-kappa B pathway may not be involved in HGF-induced PD-L1 expression in NSCLC. **Figure S6.** PD-L1 expression was increased in EGFR-TKIs resistant cells. **Figure S7.** A. *c-MET* Si-RNAs downregulate the c-MET expression in PC-9 and PC-9 cells. **Figure S8.** HLA-ABC expression level in PC-9 and PC-9R cells were measured by flow cytometry. **Figure S9.** Inhibition of NF-kappa B pathway slightly decreases PD-L1 expression induced by *c-MET* amplification. **Figure S10.** 293FT cells were transfected with control vector plasmid (NC), EGFR-19Del (19Del), or EGFR-T790M (T790M) mutation plasmids for 48–72 h, then treated with/without gefitinib for a further 24 h. All the cells were harvested and analysed by western blotting, RT-qPCR and flow cytometry. **Figure S11.** PC-9 and PC-9R cells remain the same sensitivity to gefitinib after deletion of PD-L1 gene. **Figure S12.** Overexpression of PD-L1 on PC-9 cells has no significant influence on EGFR expression and EGFR-TKIs sensitivity. Supplementary materials and methods.

**Additional file 2:** **Table S1.** Basic information of EGFR-TKIs resistant NSCLC patients.

**Additional file 3:** Quantitation results of Western blots.

## Data Availability

All the data generated or analyzed during this study are included in this published article and its supplementary files.

## Abbreviations

*ALK*: Anaplastic lymphoma kinase
AP-1: Activator protein
EGFR-TKIs: Epidermal growth factor receptor-tyrosine kinase inhibitors
H&E: Hematoxylin and eosin
HGF: Hepatocyte growth factor
IFN-γ: Interferon- γ
IHC: Immunohistochemistry
MAPK: Mitogen-activated protein kinase
MSI: Microsatellite instability
NSCLC: Non-small cell lung cancer
PD-1: Programmed death-1
PD-L1: Programmed death-ligand 1
PFS: Progression-free survival
(PI3K)/Akt: Phosphoinositide 3-kinase
TILs: Tumour-infiltrating lymphocytes
TMB: Tumour mutation burden
TME: Tumour microenvironment
