# Supplementary material for: EGFR-TKI resistance promotes immune escape in lung cancer via increased PD-L1 expression
Source: Mol Cancer. 2019 Nov 20;18:165. doi: 10.1186/s12943-019-1073-4 (PMC6864970; doi:10.1186/s12943-019-1073-4)
Supplement: Supplementary file 1 — Additional file 1: Figure S1. The tumor mutation burden (TMB), PD-L1, PD-1 and CTLA-4 expression in EGFR, c-MET and KRAS mutant subgroups basing on TCGA Datasheet (Lung Adenocarcinoma, PanCancer Atlas). Figure S2. PD-L1 Expression after stimulation of HGF. Figure S3. A. The percentage of DC cells in PBMC separated from health donators. Figure S4. A. IFN-γ concentration in the supernatant of co-culture systems. Figure S5. Inhibition of NF-kappa B pathway may not be involved in HGF-induced PD-L1 expression in NSCLC. Figure S6. PD-L1 expression was increased in EGFR-TKIs resistant cells. Figure S7. A. c-MET Si-RNAs downregulate the c-MET expression in PC-9 and PC-9 cells. Figure S8. HLA-ABC expression level in PC-9 and PC-9R cells were measured by flow cytometry. Figure S9. Inhibition of NF-kappa B pathway slightly decreases PD-L1 expression induced by c-MET amplification. Figure S10. 293FT cells were transfected with control vector plasmid (NC), EGFR-19Del (19Del), or EGFR-T790M (T790M) mutation plasmids for 48–72 h, then treated with/without gefitinib for a further 24 h. All the cells were harvested and analysed by western blotting, RT-qPCR and flow cytometry. Figure S11. PC-9 and PC-9R cells remain the same sensitivity to gefitinib after deletion of PD-L1 gene. Figure S12. Overexpression of PD-L1 on PC-9 cells has no significant influence on EGFR expression and EGFR-TKIs sensitivity. Supplementary materials and methods. [file 12943_2019_1073_MOESM1_ESM.docx]

**EGFR-TKI Resistance Promotes Immune Escape in Lung Cancer via Increased PD-L1 Expression**

**Shunli Peng^1^, Rong Wang^1^, Xiaojuan Zhang^1^, Yueyun Ma^1^, Longhui Zhong^1^, Ke Li^2^, Nishiyama Akihiro^3^, Sachiko Arai^3^, Seiji Yano^3^, Wei Wang^1^**

**Additional file Figures**

**Figure S1.** **The tumor mutation burden (TMB), PD-L1, PD-1 and CTLA-4 expression in *EGFR*, *c-MET* and *KRAS* mutant subgroups basing on TCGA Datasheet (Lung Adenocarcinoma, PanCancer Atlas).** According to the mutation models, patients were divided into 6 subgroups (*EGFR*^+^ (N=68), *EGFR*^+^*/KRAS^+^* (N=1), *c-MET^+^* (N=18), *c-MET^+^*/*KRAS^+^* (N=6), *KRAS^+^* (N=162)*,* *EGFR^+^/c-MET^+^* (N=0)). After exclusion of patients without complete information or with co-occurring genomic alterations (*EGFR^+^/KRAS^+^, c-MET^+^/KRAS^+^*), ultimately data from 235 patients including *EGFR*^+^ (N=63), *c-MET^+^* (N=18) and *KRAS^+^* (N=154) were analyzed. Bars indicate SE; ***P* < 0.05; *** P* < 0.01.


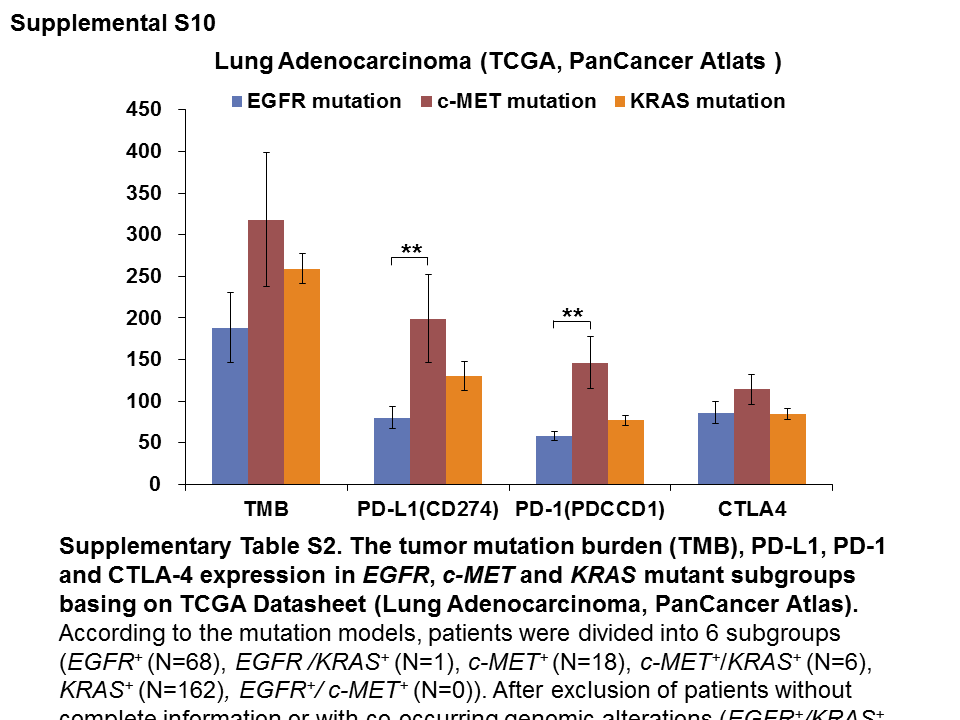


**Figure S2. PD-L1 Expression after stimulation of HGF.** PC-9 and HCC827 cells were treated with HGF (50 ng/L) for different periods of time, then harvested and measured by flow cytometry to evaluate the expression of PD-L1.

**
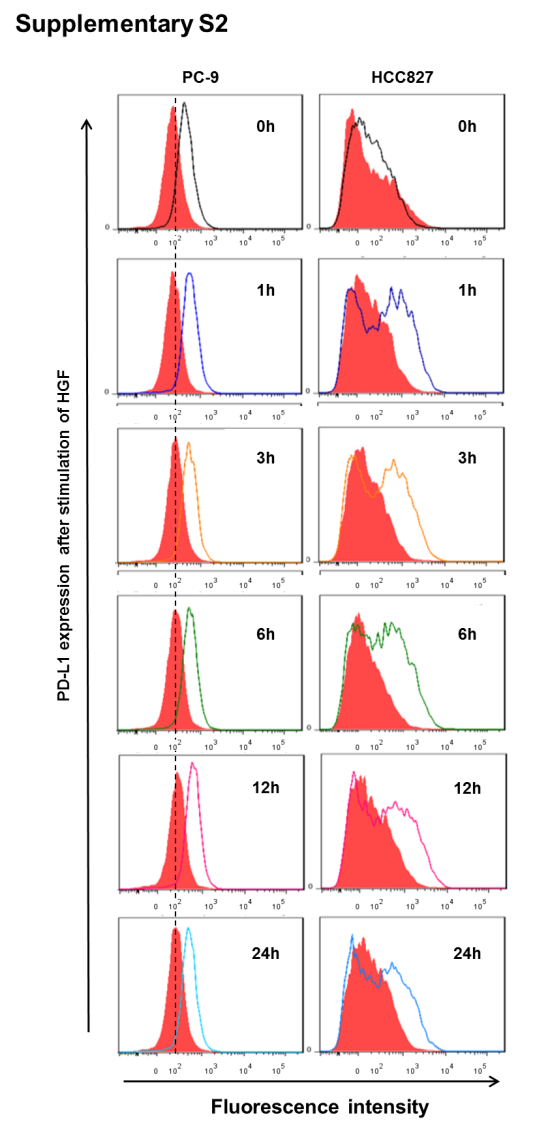
**

**Figure S3. A.The percentage of DC cells in PBMC separated from health donators.** CD1a^+^CD83^+^HLA-DR^+^ cells were marked as DC cells. **B and C.** The percentage of CD8^+^ cells in CD3^+^ or PBMC cells. PBMC cells were co-cultured with/without HCC827 cells at an effector/target cell ratio = 6:1for 72 hours, then all the cells were collected and measured by flow cytometry. **D. The HLA-ABC expression level in HCC827 cells.** HCC827 cells were treated with/without HGF(50 ng/ml) for 5 hours then harvested and measured by flow cytometry.

**
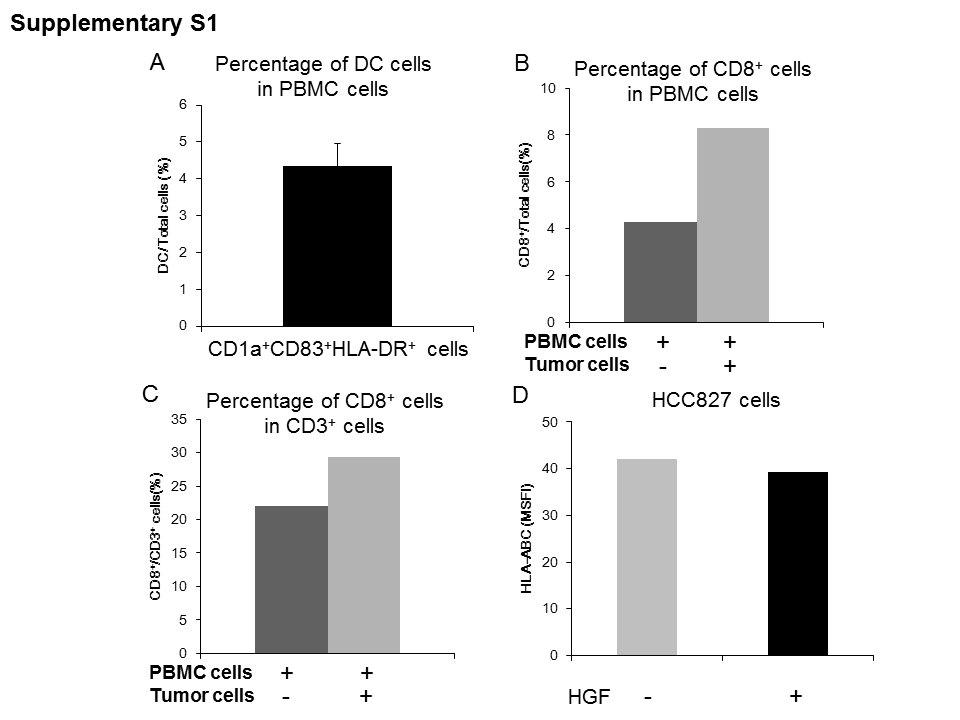
**

**
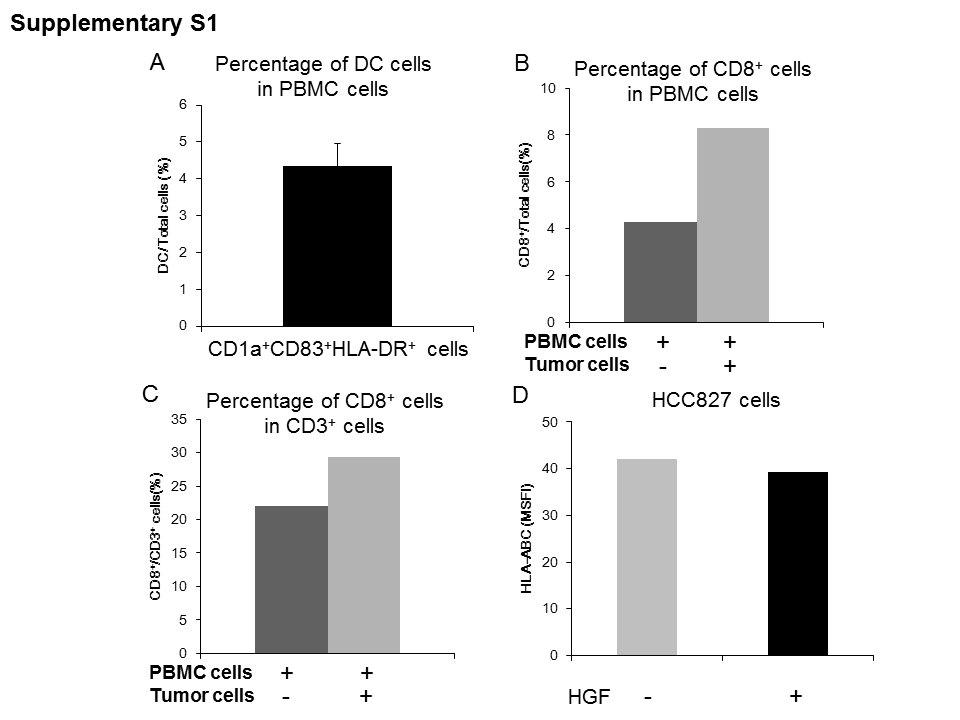
**

**Figure S4. A. IFN-γ concentration in the supernatant of co-culture systems.** HCC827 cells were pre-treated with or without HGF (50 ng/L), anti-PD-L1 antibodies (10 μg/mL) or control IgG for an hour. Then all the cells were co-cultured with human PBMC for further 72 hours at an effector/target cell ratio = 6:1. The concentration of IFN-γ in the supernatant was detected by human IFN-γ ELISA assay (multi sciences company, EK1802). Bars indicate SD,* *P* < 0.05; *** P* < 0.01. **B.** Percentage of CD3^-^CD16^+^CD56^+^ and CD3^+^CD16^+^CD56^+^ in PBMC cells. Bars indicate SD.


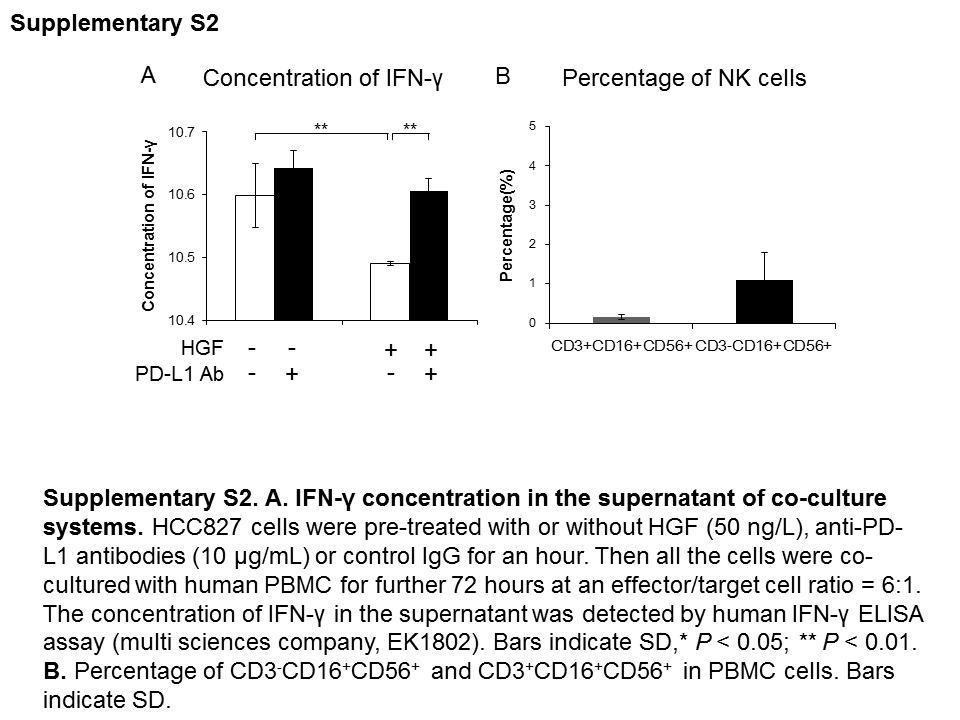


**Figure S5. Inhibition of NF-kappa B pathway may not be involved in HGF-induced PD-L1 expression in NSCLC.** PC-9/HCC827 cells were pre-treated with/without IMD 0354 (NF-kappa B pathway inhibitor, 1 μmol/L) for 19h, then HGF was/was not added at final concentration of 50 ng/ml. All the cells were cultured for further 5 hours, then harvested and measured by flow cytometry. Bars indicate SD; **P* < 0.05; *** P* < 0.01.


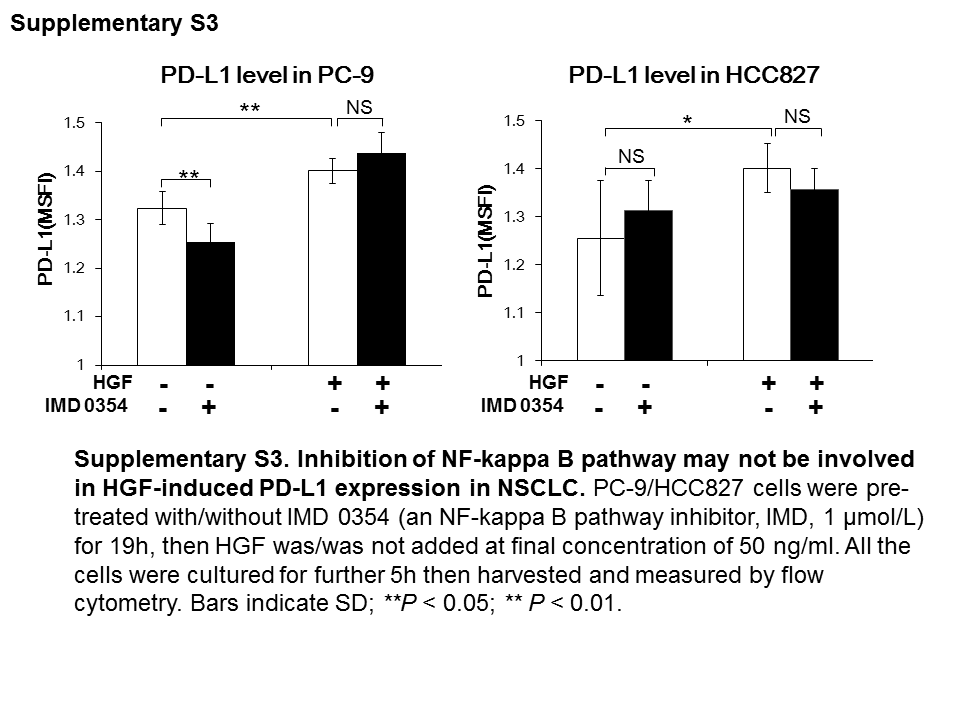


**Figure S6. PD-L1 expression was increased in EGFR-TKIs resistant cells.** PC-9 cells were exposed in increasing concentrations of gefitinib over 6 months to culture EGFR-TKIs resistant cells and the resistant clones were picked from gefitinib-resistant PC-9 cells. The resistant clones and PC-9 cells were harvested and analyzed by western blotting.

**
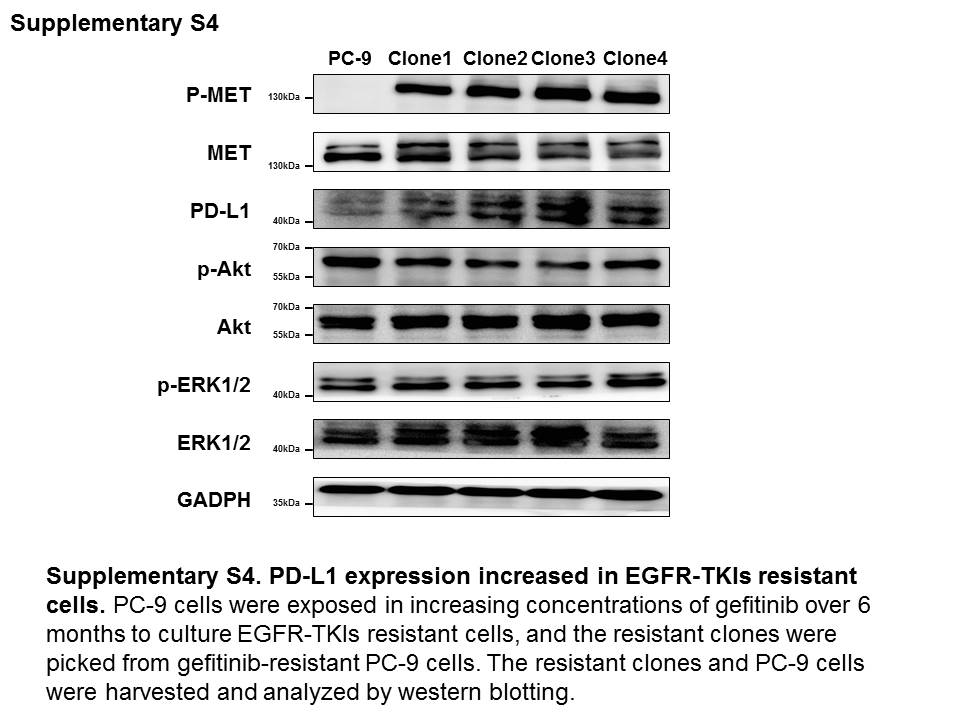
**

**Figure S7. A. *c-MET* Si-RNAs downregulate the c-MET expression in PC-9 and PC-9 cells.** PC-9 and PC-9R cells were transfected with *c-MET* si-RNA for 72 hours, the all the cells were harvested and analyzed by western blotting. **B.** PC-9 and PC-9R cells were treated with or without c-MET siRNA for 48 hours, and cultured with or without treatment of gefitinib (1 μmol/L). After further 12 hours, cells were collected and PD-L1 expression was examined by flow cytometry.

**
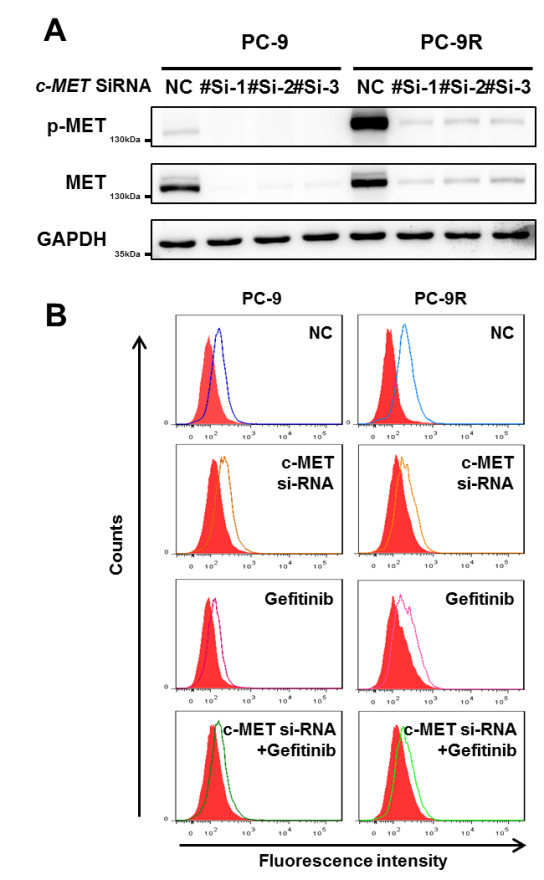
**

**Figure S8.** HLA-ABC expression level in PC-9 and PC-9R cells were measured by flow cytometry.


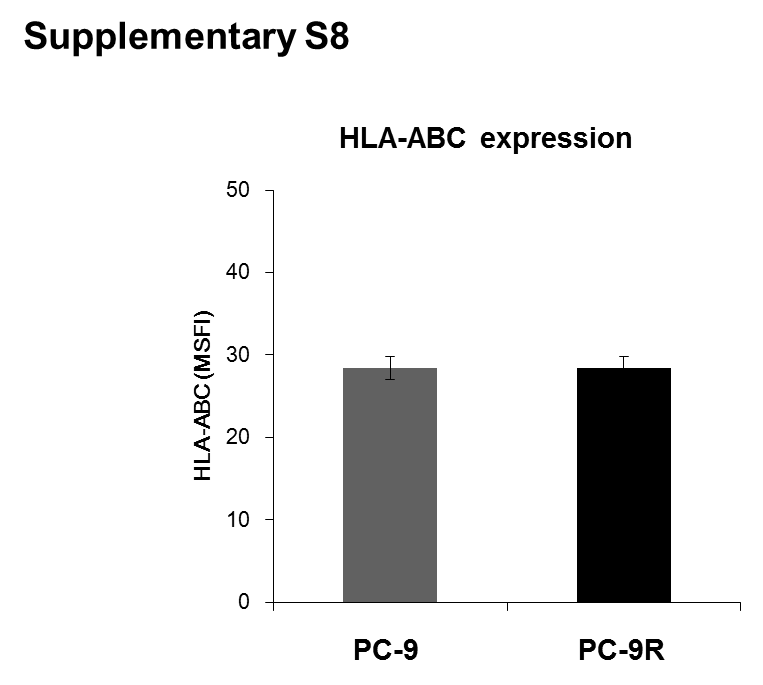


**Figure S9. Inhibition of NF-kappa B pathway slightly decreases PD-L1 expression induced by *c-MET* amplification.** PC-9 and PC-9R cells were pre-treated with IMD 0354 (IMD, 1μmol/L) for 24h, then all the cells were harvested and measured by flow cytometry. ). Bars indicate SD; ** P < 0.01.


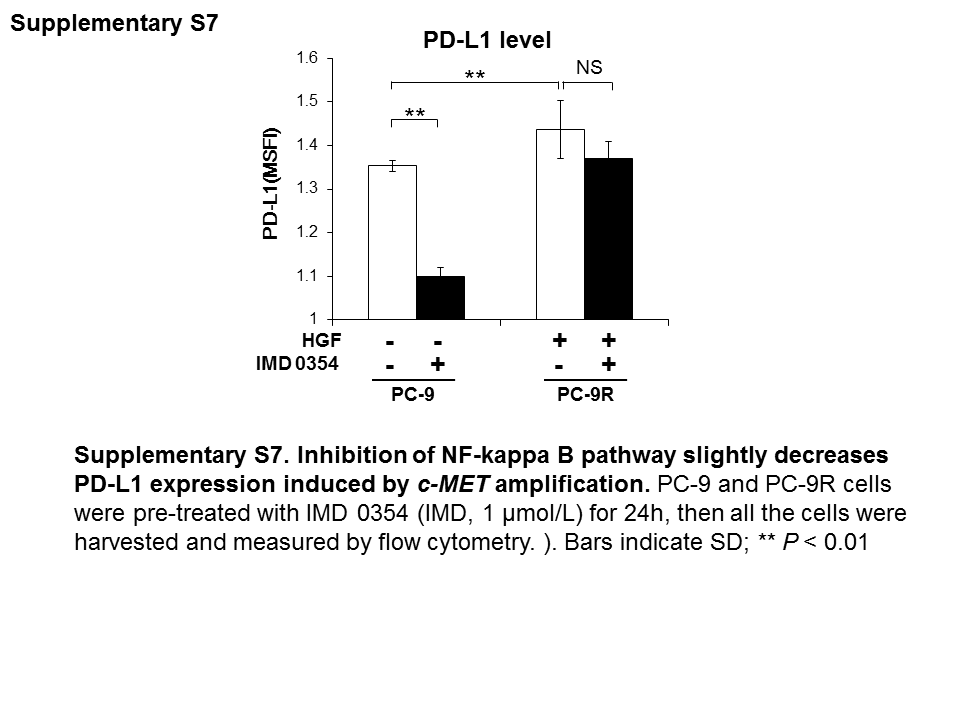


**Figure S10.** 293FT cells were transfected with control vector plasmid (NC), EGFR-19Del (19Del), or EGFR-T790M (T790M) mutation plasmids for 48-72 hours, then treated with/without gefitinib for a further 24 hours. All the cells were harvested and analysed by western blotting, RT-qPCR and flow cytometry.

**
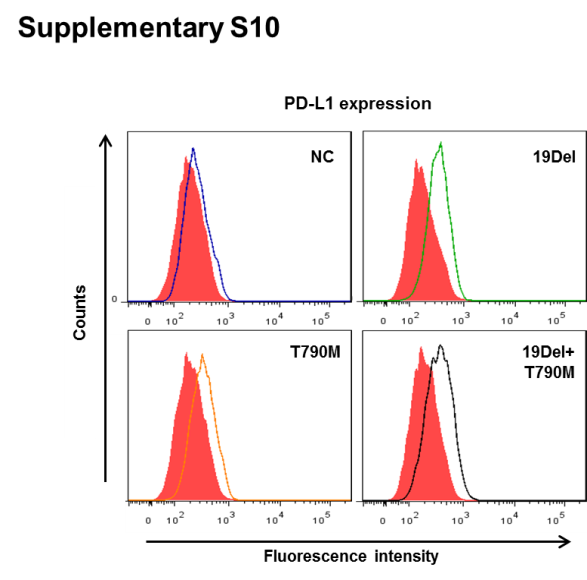
**

**Figure S11.** **PC-9 and PC-9R cells remain the same sensitivity to gefitinib after deletion of PD-L1 gene.** PC-9 ^PD-L1+^, PC-9R ^PD-L1+^, PC-9 ^PD-L1-^, PC-9R ^PD-L1-^ cells were treated with various concentrations of gefitinib after 72 hours and the growth condition of cells were measured by MTT assay. Bars indicate SD. **
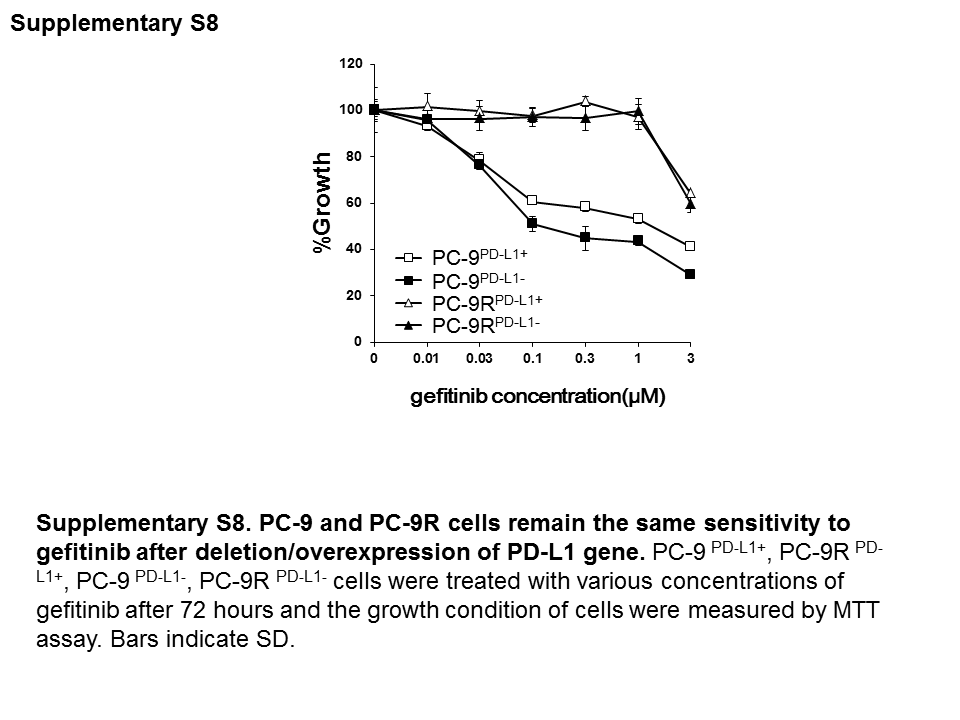
**

**Figure S12.** **Overexpression of PD-L1 on PC-9 cells has no significant influence on EGFR expression and EGFR-TKIs sensitivity.** PC-9 cells were transfected with/without PD-L1 overexpression plasmid or control vector for 72 hours, then all the cells were harvested for western blotting and MTT assay. Bars indicate SD.


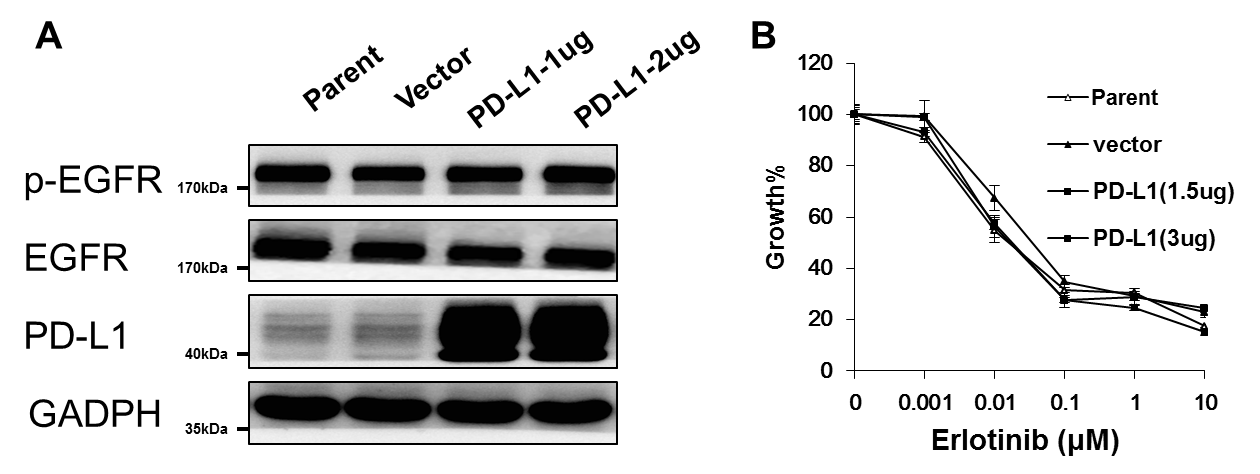


**Supplementary materials and methods**

**Reverse transcription and quantitative real-time PCR**

Total RNA was isolated from cells using RNAiso Plus (Takara, Dalian, China), and reverse-transcribed into cDNA using the PrimeScript™ RT Master Mix (TaKaRa, Dalian, China) according to the manufacturer's instructions. Quantitative real-time PCR (qRT-PCR) was performed using the Roche Light Cycler 480 (LC480) system (Roche Diagnostics, Meylan, France) with a SYBR® Premix Ex Taq™ (Tli RNaseH Plus) (TaKaRa, Dalian, China). Oligonucleotide primers used for *c-MET, PD-L1* and *GAPDH* (internal control) were as follows: c-MET: 5'-CCATCCAGTGTCTCCAGAAGTG-3' (sense); 5'-TTCCCAGTGATAACCAGTGTGTAG-3' (antisense); GAPDH:5'-AGAAGGTGGGGCTCATTTG-3' (sense); 5'-AGGGGCCATCCACAGTCTTC-3' (antisense); PD-L1:5’-CAATGTGACCAGCACACTGAGAA-3’ (sense); 5’-GGCATAATAAGATGGCTCCCAGAA-3’ (antisense). All reactions were performed in triplicate for each sample. Cycle threshold (Ct) values of *c-MET* cDNA were normalized to GAPDH using the -2^ΔΔCt^ method.

**Immunohistochemistry (IHC) and terminal dUTP nick-end labeling (TUNEL) assays**

IHC staining and TUNEL assay were performed as described[[1](#_ENREF_1)]. The PD-L1 IHCC 22C3 pharmDx assay was used to diagnose PD-L1 expression status in clinical specimens. Tumor Proportion Score (TPS= #PD-L1 positive tumor cells/ Total# of PD-L1 positive + PD-L1 negative tumor cells) were used to evaluated the expression status of PD-L1. TPS<1%, 1-49%, ≥50%, refer to No expression, Low expression and High expression of PD-L1, respectively. Anti-PD-L1 (E1L3N) and anti-Ki67 (9027) antibodies (Cell Signaling Technology) were used in the detection of xenograft samples. The secondary antibodies were rabbit anti-mouse AlexaFlour-488 secondary antibody (Abcam) or peroxidase-conjugated secondary antibodies (SP-9000; Zhongshan Gold Bridge, Guangzhou, China). Samples were visualized using a fluorescence microscope (Olympus IX70-S8F2; Olympus Optical Co., Ltd., Tokyo, Japan).

**Flow cytometric analysis**

For this study, BD FACS Calibur flow cytometry with Flow Jo v.9 and CellQuest software (Becton Dickinson, San Jose, CA) were used. Anti-PD-L1 (E1L3N, 1:400 dilution), and secondary antibodies were anti-rabbit IgG (H+L), and F(ab')2 Fragment (Alexa Fluor®555 Conjugate,#4413), which were purchased from Cell Signaling Technology. The mean fluorescence intensity (MFI) and mean specific fluorescence intensity (MSFI) was used to evaluate the PD-L1 expression level, and MSFI is calculated as the ratio of MFI of anti-PD-L1 antibody to that of control antibody[[2](#_ENREF_2)].

**Analysis of TCGA datasheet**

The TCGA datasheet of Lung Adenocarcinoma (PanCancer Atlats, provisional) were retrieved from cBioProtal (http://www.cbioportal.org/).

A total of 517 NSCLC tumors in PanCancer Atlats datasheet and 503 NSCLC tumors with detail information about mutation load and mRNA expression levels were analyzed. Basing on mutation status of *EGFR*, *c-MET* and *KRAS* genes, patients in PanCancer Atlats datasheet were divided into *EGFR*^+^ (N=68), *EGFR* */KRAS^+^* (N=1), *c-MET^+^* (N=18), *c-MET^+^*/*KRAS^+^* (N=6), *KRAS^+^* (N=162)*,* subgroups for further study. No *EGFR^+^/ c-MET^+^* (N=0) mutant model was found in this cohort. All data were presented as the mean ± standard deviation (SD) or mean ± standard error (SE), and differences between the means were examined by T test or one-way ANOVA using statistical software (SPSS, version 20, IBM Corp., Armonk, USA). A value of P < 0.05 was considered to indicate a statistically significant difference. All experiments were performed at least three times.

Reference

1. Liu T, Sun Q, Li Q, Yang H, Zhang Y, Wang R, Lin X, Xiao D, Yuan Y, Chen L, Wang W: **Dual PI3K/mTOR inhibitors, GSK2126458 and PKI-587, suppress tumor progression and increase radiosensitivity in nasopharyngeal carcinoma.** *Mol Cancer Ther* 2015, **14:**429-439.

2. Wang W, Nishioka Y, Ozaki S, Jalili A, Abe S, Kakiuchi S, Kishuku M, Minakuchi K, Matsumoto T, Sone S: **HM1.24 (CD317) is a novel target against lung cancer for immunotherapy using anti-HM1.24 antibody.** *Cancer Immunol Immunother* 2009, **58:**967-976.
