# Supplementary material for: EGFR-TKI resistance promotes immune escape in lung cancer via increased PD-L1 expression
Source: Mol Cancer. 2019 Nov 20;18:165. doi: 10.1186/s12943-019-1073-4 (PMC6864970; doi:10.1186/s12943-019-1073-4)
Supplement: Supplementary file 2 — Additional file 2: Table S1. Basic information of EGFR-TKIs resistant NSCLC patients. [file 12943_2019_1073_MOESM2_ESM.pdf]

EGFR-TKI Resistance Promotes Immune Escape in Lung Cancer via Increased PD-L1 Expression

Shunli Peng[1], Rong Wang[1], Xiaojuan Zhang[1], Yueyun Ma[1], Longhui Zhong[1], Ke Li[2], Nishiyama Akihiro[3], Sachiko Arai[3], Seiji Yano[3], Wei Wang[1]

Supplementary Table S1

Supplementary Table S1. Basic information of EGFR-TKIs resistant NSCLC patients

Supplemental Table. S1

Basic information of EGFR-TKIs resistant NSCLC patients

Basic information				Tumor type and TNM stage								Mutant and Resistant informatiion		
Sample ID (biopsies)	Female / Male	Age at diagnosis	Smoking History	Cancer Type	Biopsy Sample Type	American Joint Committee on Cancer Publication Version Type						Mutation Type	EGFR-TKIs therapy type	Resistant Mechanisms
①H07-01366	Male	65	NO	Adenocarcinoma	Primary Tumor	6th	IIIA	T2	N2	M0		EGFR mutant	Gefitinib	Unknown
②H09-08273	Female	71	NO	Adenocarcinoma	Primary Tumor	7th	IIIA	T2	N2	M0		EGFR mutant	Gefitinib	Unknown
③H12-03382	Female	60	NO	Adenocarcinoma	Primary Tumor	7th	IIIA	T3	N1	M0		EGFR mutant	Gefitinib	Unknown
④H10-02317	Female	52	NO	Adenocarcinoma	Primary Tumor	7th	IIA	T2	N1	M0		EGFR mutant	Erlotinib	Unknown
⑤H03-06169	Female	57	NO	Adenocarcinoma	Primary Tumor	6th	IB	T2	N0	M0		EGFR mutant	Gefitinib	Unknown
⑥H09-05014	Male	69	NO	Adenocarcinoma	Primary Tumor	7th	IIIA	T2	N2	M0		EGFR mutant	Erlotinib	Unknown
⑦H08-03231	Female	78	NO	Adenocarcinoma	Primary Tumor	7th	IIIA	T2	N2	M0		EGFR mutant	Erlotinib	Unknown
⑧H08-03316	Male	64	NO	Adenocarcinoma	Primary Tumor	7th	IV	T2a	N2	M1a		EGFR mutant	Erlotinib	T790M
⑨H09-06235	Female	69	NO	Adenocarcinoma	Primary Tumor	7th	IA	T1a	N0	M0		EGFR mutant	Erlotinib	T790M
⑩H12-01756	Female	67	NO	Adenocarcinoma	Primary Tumor	7th	IIIA	T1b	N2	M0		EGFR mutant	Erlotinib	Unknown
⑪H12-08282	Female	52	YES	Adenocarcinoma	Primary Tumor	7th	IV	T1a	N2	M1b		EGFR mutant	Gefitinib	Unknown
⑫H16-07183	Male	85	YES	Adenocarcinoma	Primary Tumor	7th	IV	T1b	N2	M1b		EGFR mutant	Gefitinib	Unknown
⑬H15-03460	Male	74	NO	Adenocarcinoma	Primary Tumor	7th	IV	T2	N2	M1b		EGFR mutant	Gefitinib	Unknown
⑭H12-08117	Female	62	NO	Adenocarcinoma	Primary Tumor	7th	IB	T2a	N0	M0		EGFR mutant	Gefitinib	Unknown
⑮H16-04430	Female	71	NO	Adenocarcinoma	Lymph-node	7th	IV	T3	N3	M1b		EGFR mutant	Gefitinib	T790M
