# Supplementary figures and images for: EGFR-TKI resistance promotes immune escape in lung cancer via increased PD-L1 expression
Source: Mol Cancer. 2019 Nov 20;18:165. doi: 10.1186/s12943-019-1073-4 (PMC6864970; doi:10.1186/s12943-019-1073-4)

**Additional file for quantitation of WB**


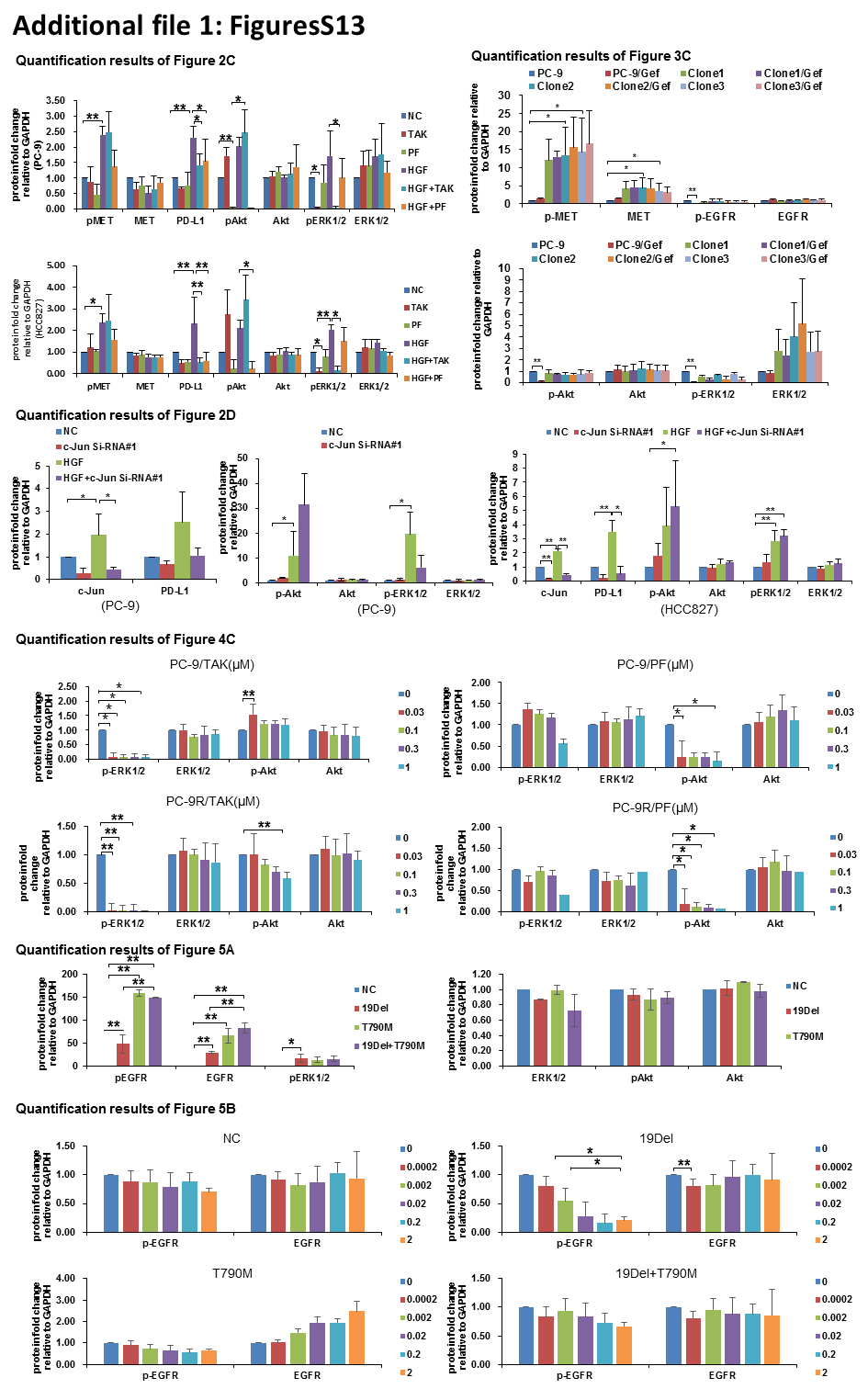

Supplement: Supplementary file 3 — Additional file 3: Quantitation results of Western blots. [file 12943_2019_1073_MOESM3_ESM.docx]
